# Extracellular Vesicles as Molecular Regulators of Viral Infection: Implications for COVID-19 and Future Viral Pandemics

**DOI:** 10.3390/cimb48090924

**Published:** 2026-09-10

**Authors:** Saugata Dutta, Sauradeep Dutta, Yohan Han, Yin Zhu, Sultan Almuntashiri, Payaningal R. Somanath, S. Priya Narayanan, Shaheen Islam, Xiaoyun Wang, Duo Zhang

**Affiliations:** 1 Clinical and Experimental Therapeutics, College of Pharmacy, University of Georgia and Charlie Norwood VA Medical Center, Augusta, GA 30912, USA; 2 Yogananda School of AI, Computer and Data Science, Shoolini University, Solan 173229, Himachal Pradesh, India; 3 Institute of Wonkwang Medical Science and Department of Microbiology, Wonkwang University School of Medicine, Iksan 54538, Jeonbuk, Republic of Korea; 4 Department of Clinical Pharmacy, College of Pharmacy, University of Ha’il, Ha’il 55473, Saudi Arabia; 5 Division of Pulmonary, Critical Care & Sleep Medicine, Medical College of Georgia, Augusta University, Augusta, GA 30912, USA; 6 Department of Medicine, Medical College of Georgia, Augusta University, Augusta, GA 30912, USA

**Keywords:** extracellular vesicles, COVID-19, SARS-CoV-2, ACE2, TMPRSS2, NF-κB, NLRP3 inflammasome, biomarkers, future viral pandemics

## Abstract

Coronavirus disease 2019 (COVID-19), caused by Severe Acute Respiratory Syndrome Coronavirus 2 (SARS-CoV-2), exposed critical gaps in global preparedness for rapidly evolving viral pandemics. Though current antiviral treatments have shown some efficacy in managing COVID-19, substantial limitations, such as inefficient targeted drug delivery, aberrant host immune responses, and inadequate preparedness for future viral pandemics, were also evident. These challenges underscore the urgent need for target-specific, precise and efficient therapeutic strategies to modulate viral entry and immune dysregulation. Extracellular vesicles (EVs), cell-released cargo-carrying nanoscale vesicles, can both facilitate and inhibit viral infection depending on their cargo composition. Thus, endogenous EVs can promote or inhibit COVID-19 pathogenesis by modulating critical pathways, including angiotensin-converting enzyme 2 (ACE2)-mediated viral entry, transmembrane protease serine 2 (TMPRSS2)- dependent spike protein activation, nuclear factor kappa B (NF-κB)-driven inflammatory signaling, and NLRP3 inflammasome activation. In contrast, engineered EVs, such as ACE2-expressing EVs, mesenchymal stem cell-derived EVs and microRNA-enriched EVs, have therapeutic potential as they can facilitate antiviral defense through immune modulation, viral neutralization and suppression of cytokine storm. Moreover, EV-associated nucleic acids, proteins and lipids can act as biomarkers for disease detection and severity stratification. A deeper understanding of EV-virus mechanistic insights can enhance preparedness for similar viral diseases. In this review, we provide a comprehensive analysis of the molecular mechanisms underlying EV-virus interactions highlighting the therapeutic and diagnostic potential of EVs in tackling COVID-19 and future viral pandemics.

## 1. Introduction

The global community faced significant challenges during the COVID-19 pandemic, identifying the limitations in the rapid development of targeted and effective therapeutic and diagnostic strategies [1]. Vaccines and antiviral therapies have substantially reduced COVID-19-related morbidity and mortality [2,3]. However, challenges persist and severe cases of COVID-19 can still result in high mortality or long-term health consequences that significantly impact quality of life [4]. The limited efficacy of vaccines and anti-viral therapies is attributed to various factors, including immune evasion, viral mutations, dysregulated host immune responses, and lack of tissue-specific drug delivery [5,6,7,8]. Collectively, these limitations underscore the critical need for advanced, biologically targeted, and adaptable approaches to address both present and emerging viral pathogens.

Extracellular vesicles (EVs) are nanoscale lipid bilayer-enclosed particles secreted by virtually all cell types and play essential roles in mediating intercellular communication [9]. EVs transport a diverse array of bioactive cargo, including proteins, lipids, messenger RNAs, and microRNAs (miRNAs), thus influencing recipient cell function at the molecular level [10]. Unlike synthetic drug delivery systems, EVs possess intrinsic biocompatibility, low immunogenicity, and natural targeting capabilities, making them attractive candidates for therapeutic and diagnostic applications [11,12].

EVs not only act as passive carriers of biomolecules, but also actively participate in viral pathogenesis [13]. Emerging evidence demonstrates that EVs modulate multiple molecular pathways central to SARS-CoV-2 infection and pathogenesis of COVID-19 [14,15]. These include ACE2-mediated viral entry, TMPRSS2-dependent spike protein priming, endosomal trafficking pathways, and several immune signaling pathways, including NF-κB, JAK/STAT, and NLRP3 inflammasome activation [16,17,18]. Through these mechanisms, EVs can either facilitate viral dissemination or contribute to antiviral defense, depending on their cellular origin and cargo composition [19].

In addition to their role in pathogenesis, EVs have shown significant promise as therapeutic platforms [11]. Engineered EVs can be designed to function as viral decoys, targeted drug delivery vehicles, or immunomodulatory agents [11,14]. EV-associated molecular signatures provide valuable opportunities for non-invasive diagnosis of disease status and progression [20]. Given their versatility and mechanistic involvement in viral biology, EVs represent a compelling platform for both current COVID-19 management and preparedness for evolving viral pathogens [10,14].

This review aims to provide a comprehensive and mechanistically focused overview of EV biology in the context of COVID-19, focusing on the role of EVs in viral infection, immune modulation, therapeutic development, and diagnostic innovation for COVID-19 and future viral pandemics. Unlike earlier reviews that primarily summarized EV biology, COVID-19-associated EV biomarkers, or therapeutic applications [14,15], this review integrates EV–virus interactions with specific molecular pathways involved in viral entry, intracellular trafficking, inflammatory signaling, and inflammasome activation, and then connects these mechanisms to emerging therapeutic and diagnostic strategies. Particular emphasis is placed on distinguishing mechanistic evidence from hypothesis-driven applications and on assessing the translational barriers that must be addressed before EV-based technologies can enter routine clinical use.

## 2. COVID-19

Coronavirus disease 2019 (COVID-19), caused by SARS-CoV-2, is a transmissible respiratory disease with pulmonary and systemic manifestations [21]. SARS-CoV-2 is an enveloped, positive-sense RNA virus that is composed of four major structural proteins—spike (S), envelope (E), membrane (M), and nucleocapsid (N) proteins [22]. The S glycoprotein mediates host cell entry through receptor recognition and membrane fusion [22]. Infection primarily targets the respiratory system, leading to clinical outcomes ranging from mild respiratory disease to acute lung injury (ALI), including its severe form, acute respiratory distress syndrome (ARDS) which is associated with severe disease and to mortality [23,24].

SARS-CoV-2 continues to evolve, with variants and sublineages showing changes in transmissibility, receptor binding, and immune escape [5]. Though vaccination and antiviral therapy have substantially reduced disease burden, challenges exist in patients with severe disease and in the setting of viral evolution and dysregulated host immune responses [25]. These features are directly relevant to EV biology because EVs can modulate virus–host interaction, inflammatory signaling, immune responses, and biomarker profiles [26], providing the mechanistic context for the subsequent sections of this review.

Extracellular vesicles (EVs) have emerged as important mediators of intercellular communication during viral infections and may have particular relevance to COVID-19 because of their ability to reflect and influence disease-associated cellular responses [27]. In SARS-CoV-2 infection, EVs have been investigated as potential carriers of host- and virus-associated molecules, as well as potential indicators of disease status and severity [27,28]. Their presence in biological fluids and their capacity to transport diverse molecular cargos also make EVs attractive candidates for both diagnostic and therapeutic applications [26,28]. Collectively, these multifaceted properties provide a rationale for examining the involvement of EVs in COVID-19 pathogenesis, their potential utility as biomarkers, and their emerging therapeutic applications.

## 3. Extracellular Vesicles

### 3.1. Biological Function and Cargo Composition

**Figure 1:**
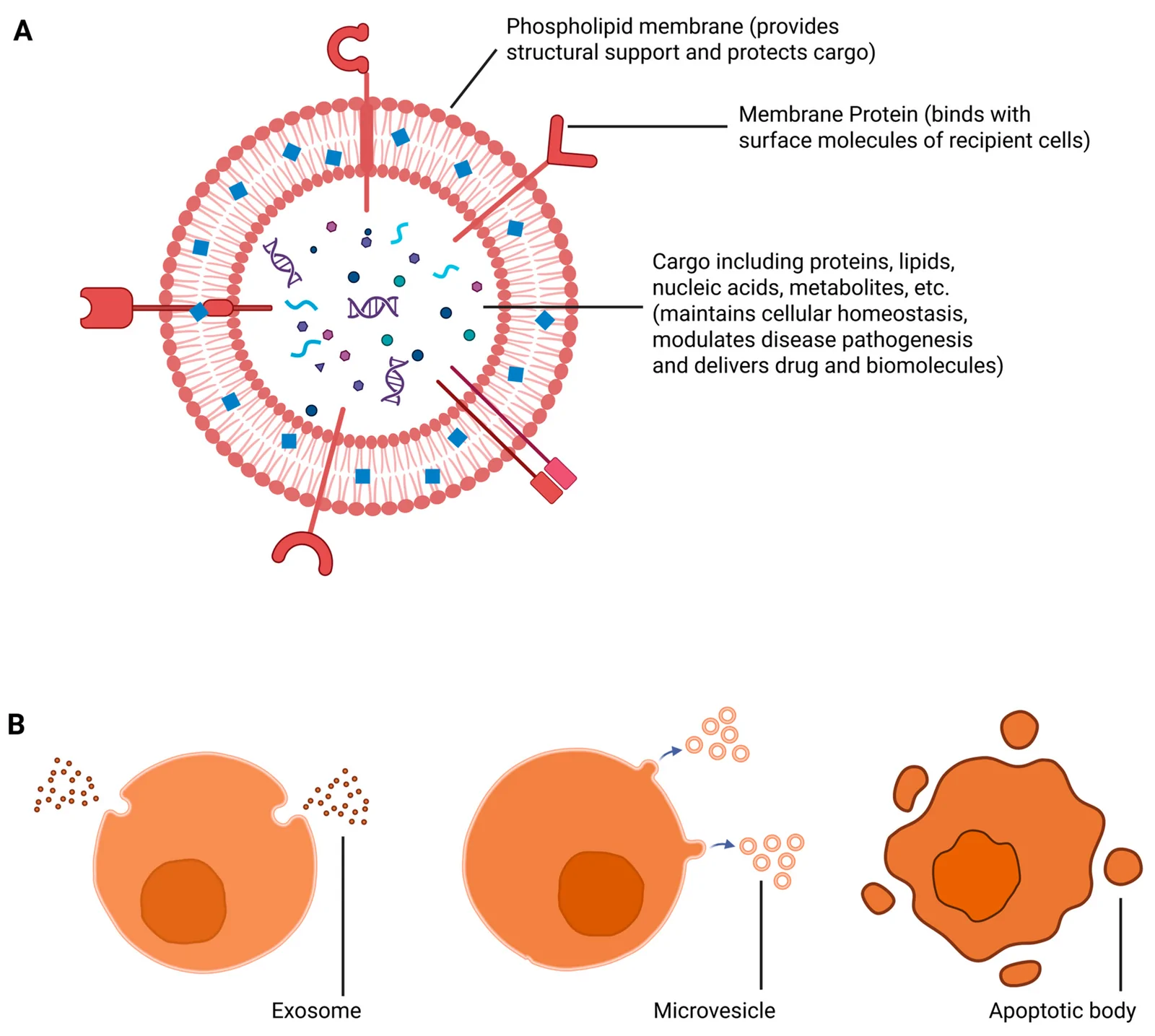
General structure and classification of EVs: (**A**) Schematic diagram of the cross-section of an EV portrays the structure of EVs showing representative membrane components and cargo, along with their major biological functions. (**B**) Schematic diagram of different types of EVs shows how exosomes, microvesicles and apoptotic bodies are generated. Created in BioRender. Dutta, S. (2026) https://BioRender.com/fhke0nh.

EVs are lipid-bilayer-enclosed extracellular particles released by cells and involved in intercellular communication [29]. They are naturally released from all three domains of life, viz., bacteria, archaea, and eukarya [30,31]. Virtually, all mammalian cells release EVs [32]. EVs can contain proteins, nucleic acids, lipids, metabolites, chemical and structural components, and other bioactive molecules (Figure 1A) and transfer them to recipient cells throughout the body [29]. As of 11 May 2026, Vesiclepedia has recorded 3481 studies on EVs that found 566,911 proteins, 27,692 mRNAs, 22,858 microRNAs (miRNAs), and 3839 lipids in EVs from 56 different species [33]. Their cargo composition reflects the producing cell and its physiological or pathological state and can alter recipient-cell signaling after uptake [34]. Through the transfer of bioactive cargo, EVs can play crucial roles in maintaining cellular homeostasis and modulating disease pathogenesis [29]. Their membrane composition and associated proteins contribute to EV uptake and interactions with recipient cells, while their membrane-enclosed cargo can be detected in biological fluids, supporting their investigation as therapeutic carriers and liquid-biopsy biomarkers [34,35].

### 3.2. Biogenesis and Classification

According to the presumed biogenesis and size, EVs have traditionally been categorized into three groups: exosomes, microvesicles and apoptotic bodies (Figure 1B) [36]. Exosomes are generally described as small EVs (30–100 nm) that are generated at the endosome by inward budding of the membrane [37,38], whereas microvesicles are 100–1000 nm vesicular structures that are generated from the plasma membrane by outward blebbing [36]. Apoptotic bodies (1–5 µm) are generated during the late stages of apoptosis [36]. However, the current International Society for Extracellular Vesicles (ISEV) recommendations emphasize the umbrella term “extracellular vesicle” and caution against assigning biogenesis-based terms, such as “exosome” or “microvesicle”, unless the specific biogenesis pathway has been demonstrated [34]. Operational descriptors based on size, density, molecular composition, or cellular origin are therefore preferred when biogenesis of an EV population cannot be established [34]. For plasma membrane-derived vesicles, ISEV2023 discourages the use of the term “microvesicle” because the term has historically been used inconsistently to describe large EVs or broader EV populations [34]. In particular, terms such as small EVs (sEVs) and large EVs (lEVs) may be used as operational descriptors based on size, provided that the size criteria are clearly defined and their inherent overlap is acknowledged [34]. This updated terminology is important when interpreting studies of EVs in COVID-19 because the term “exosome” has been used broadly in the literature, sometimes without definitive evidence of the corresponding biogenesis pathway [34,39].

### 3.3. EVs as a Drug Delivery System

EVs have several properties that support their investigation as a drug-delivery system (DDS), including a lipid-bilayer membrane, endogenous cargo-loading mechanisms, and the ability to transfer drugs, proteins and nucleic acids to recipient cells [40]. EVs can be engineered to carry therapeutic RNA, proteins, peptides, or small molecules and can be modified to enhance cellular targeting [41]. However, claims of superior targeting or efficacy compared with synthetic delivery systems remain context-dependent and require direct comparative evaluation [42]. These properties provide the rationale for the therapeutic strategies discussed in Section 5, but their clinical translation is constrained by limited cargo-loading efficiency, manufacturing and quality-control challenges, variable biodistribution, and potential off-target effects [40,42,43].

## 4. Molecular Mechanisms of EV–SARS-CoV-2 Interactions

### 4.1. Functional Interplay Between Viruses and EVs

Both viruses and EVs are primarily submicron-sized biological particles, released by living cells, and can transfer bioactive molecules and genetic materials [44]. Similarities also exist between the cellular machinery involved in the biogenesis of viruses and EVs, resulting in a blurred distinction between certain viruses and EV subtypes [44]. Among the different types of EVs, exosomes almost perfectly resemble the virions of enveloped viruses, including retroviruses [30]. Viruses and EVs may utilize the same receptors for cellular entry [44]. Viruses can also exploit EV biogenesis and secretion pathways to facilitate viral dissemination and modulate recipient-cell responses, as demonstrated for pathogens such as hepatitis C virus (HCV), and Epstein–Barr virus (EBV) and herpesviruses [45,46,47]. Furthermore, virus-infected cells generate EVs that exhibit modified physiological characteristics [44]. Rather than stimulating immune responses, these altered EVs attenuate immune reactions [48]. Thus, EVs not only facilitate viral transmission but also shield viruses from immune surveillance [49]. However, the extent to which EVs directly mediate infectious virus transmission versus transport viral components remains dependent on the virus and experimental context, and these mechanisms are not uniformly established across viral infections. This important attribute of EVs can be leveraged to inhibit viral transmission through the development of engineered EVs.

### 4.2. ACE2-Mediated Viral Entry and EV Decoy Function

SARS-CoV-2 enters the host cell when the receptor-binding domain (RBD) of the viral spike (S) protein binds to the host angiotensin-converting enzyme 2 (ACE2) receptor [16]. The ubiquitous receptor ACE2 is highly expressed in the lungs, particularly in alveolar type II (AT2) cells [50]. Thus, SARS-CoV-2 has a natural propensity to infect AT2 cells, and the virus enters the lungs specifically through the AT2 cells of the lungs [51,52]. The RBD of the viral S protein recognizes and binds to ACE2 [53]. Omicron variant (also known as the B.1.1.529 variant) of SARS-CoV-2 possesses 39 mutations in the S protein [54], 15 of which are in the RBD [6]. This unique feature has made Omicron more transmissible than any of its preceding variants and the most dominant global variant of SARS-CoV-2 [54].

**Figure 2:**
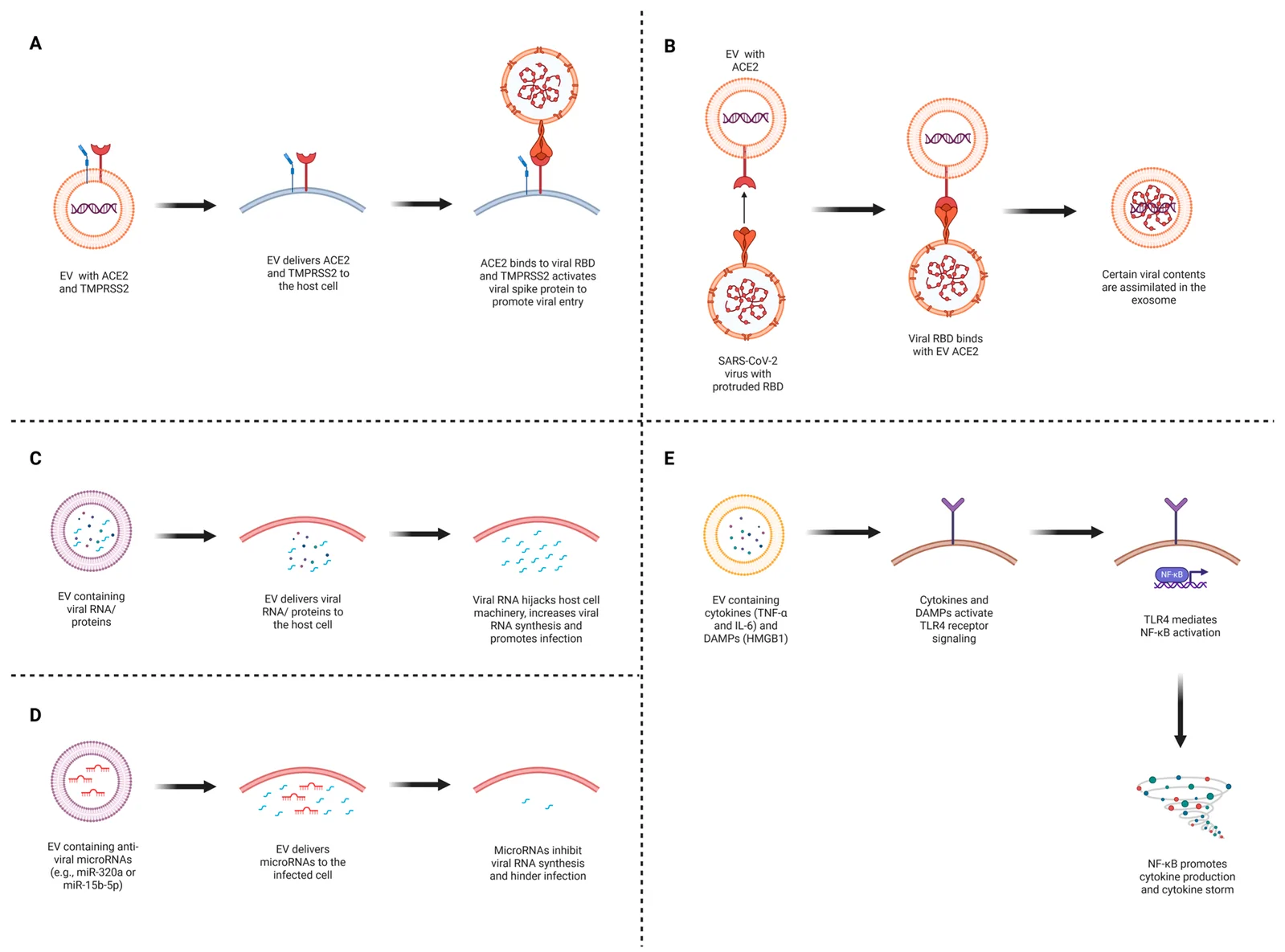
Molecular mechanisms of EV-virus interactions: (**A**) Schematic diagram of how EVs deliver ACE2 and TMPRSS2 to the host cells and promote EV-mediated viral entry. (**B**) Schematic diagram of how viral RBD fuses with EV ACE2 to assimilate certain viral contents into the EV. (**C**) Schematic diagram of how EVs transfer viral RNA/protein to the host cell, increase viral RNA synthesis and promote infection. (**D**) Schematic diagram of how engineered EVs can deliver microRNAs (e.g., miR-320a or miR-15b-5p) to the infected cells, inhibit viral RNA synthesis and hinder infection. (**E**) Schematic diagram of how EVs carry cytokines (e.g., TNF-α and IL-6) and DAMPs (e.g., HMGB1) which activate TLR4 to mediate NF-κB activation and ultimately promote cytokine production and cytokine storm. Created in BioRender. Dutta, S. (2026) https://BioRender.com/zr3kzr7.

As EVs express ACE2 on their surface, EVs can regulate viral entry dynamics in both directions [55]. When EVs deliver ACE2 to recipient cells, it can increase susceptibility to infection by increasing the availability of ACE2 receptors on host cells (Figure 2A) [55]. Moreover, interactions between ACE2-expressing EVs and SARS-CoV-2 may facilitate the incorporation of viral proteins or RNA into EVs under certain conditions (Figure 2B); thus EVs may contribute to the systemic dissemination of viral components [49]. On the other hand, ACE2-enriched EVs can function as competitive decoys and bind to the SARS-CoV-2 spike protein, thus preventing host cell-virus interaction [55]. Post-translational modifications, such as palmitoylation, enhance viral binding affinity and the neutralization potential of ACE2. Palmitoylation of ACE2 enhances its stability on the EV membrane and incorporation into EVs. Decoy efficiency is also influenced by the abundance of ACE2 displayed on the EV surface [56]. While ACE2-mediated viral binding and the decoy activity of ACE2-expressing EVs have been demonstrated experimentally, the extent to which naturally occurring ACE2-positive EVs contribute to SARS-CoV-2 entry or protection in vivo remains incompletely defined.

### 4.3. Protease Activation and Endosomal Entry Pathways

Following ACE2 binding, the viral spike protein requires proteolytic activation by host proteases, including TMPRSS2 and endosomal cathepsins [16]. EVs may influence this process by delivering proteases and modulating the local proteolytic environment (Figure 2A) [57]. Moreover, EV biogenesis shares common viral entry and trafficking pathways, particularly involving endosomal machinery such as Ras-associated binding guanosine triphosphatases (Rab GTPases), tumor susceptibility gene 101 (TSG101), ALG-2-interacting protein X (ALIX), and endosomal sorting complexes required for transport (ESCRT) [58]. These shared pathways naturally provide a mechanistic basis for viral exploitation of EV biogenesis systems to enhance viral replication and dissemination. However, the direct contribution of EV-mediated delivery of TMPRSS2 or other proteases to SARS-CoV-2 entry has not been established, and the proposed role of EV pathways in this process remains largely mechanistic or context-dependent.

### 4.4. EV-Mediated Regulation of Viral Infection

EVs play a critical role in regulating viral infection [13]. Virus-infected cell-derived EVs contain viral proteins and portions of viral RNA [13]. Depending on the viral proteins and genetic materials incorporated, EVs can both promote and hinder viral infection (Figure 2C,D) [13]. EV-mediated transfer can facilitate delivery of viral material or alter recipient-cell susceptibility and immune responses [59]. In SARS-CoV-2 infection, EV-associated viral components and vesicle-mediated transfer have been proposed to contribute to viral dissemination [60]. Although EV-associated SARS-CoV-2 components and vesicle-mediated transfer have been reported experimentally, their contribution to productive viral dissemination in vivo remains incompletely established. EVs can also carry viruses as viral clusters and can mediate ‘vesicle-mediated en bloc transmission’ of viruses. In this process, multifarious viral genomes, such as viral RNA and other viral particles, can simultaneously penetrate and cause infection in the same cell [48]. Virus-infected cells spontaneously release EVs, mostly exosomes [26]. These EVs can transport viral components to adjacent cells and induce them to facilitate further viral transmission [61]. Conversely, there is considerable potential to engineer the immune functions of EVs to inhibit the progression of COVID-19 [13]. In addition, EVs have a unique feature to influence the cellular and humoral responses based on the cargo. This powerful attribute provides EVs with a significant advantage in mitigating the hyperinflammatory effects of COVID-19 [62] and suggests their potential utility in future viral pandemics.

### 4.5. EV-Mediated Activation of NF-κB and Cytokine Storm

Hyperactivation of NF-κB-dependent inflammatory signaling pathways and cytokine storm are considered hallmarks of severe COVID-19 [63]. Infected cell-derived EVs carry pro-inflammatory mediators, including cytokines (e.g., TNF-α and IL-6) and damage-associated molecular patterns (DAMPs), such as high mobility group box 1 (HMGB1) (Figure 2E) [64]. These EV cargos can activate Toll-like receptor (TLR) signaling pathways, including TLR4-mediated NF-κB activation, thereby promoting pro-inflammatory cytokine production and cytokine storm [65]. Although EV-associated inflammatory cargos can activate NF-κB signaling in experimental models, the extent to which this EV-mediated mechanism drives cytokine storm in patients with severe COVID-19 remains to be fully established.

### 4.6. NLRP3 Inflammasome Activation

EVs play a substantial role in activating the NOD, LRR, and pyrin domain-containing protein 3 (NLRP3) inflammasome, a key mediator of inflammatory and pyroptotic reactions in COVID-19 [66]. Molecules such as mitochondrial DNA (mtDNA), viral RNA, and ROS-associated signaling molecules induce NLRP3 inflammasome assembly [67]. EVs can transfer these molecules to recipient cells and promote the assembly of the NLRP3 inflammasome [66,67]. Activation of NLRP3 can lead to caspase-1 activation, maturation of IL-1β and IL-18, and gasdermin D-mediated pyroptosis [68]. Ultimately, these factors contribute to the exacerbation of lung injury in severe COVID-19 cases. Although EV-mediated delivery of inflammasome-activating cargos has been demonstrated in experimental systems, its specific contribution to NLRP3 activation and lung injury during human SARS-CoV-2 infection remains incompletely defined.

The molecular mechanisms through which EVs regulate SARS-CoV-2 infection extend beyond a single pathway and involve coordinated effects on viral entry, intracellular trafficking, inflammatory signaling, and innate immune activation. Table 1 summarizes these key mechanistic axes together with their representative EV cargo, biological functions, and potential translational implications.

## 5. Therapeutic Potentials of EVs for COVID-19

EVs may help in maintaining the quality of the delivered drugs and delivering the drug to the target region. The lipid bilayer covering of EVs ensures a safe milieu for their inner molecules, including antibodies and cytokines, so that these molecules can maintain a strong host immunogenic response for a prolonged duration. Without the protective lipid-bilayer sheath, the inner molecules could be vulnerable to destruction while circulating in the body fluids [69]. Thus, EVs can deliver drugs, proteins, nanoparticles, miRNAs, siRNAs, etc., while retaining the quality of the cargo. Some components of EVs can promote tissue targeting, whereas some components can reduce non-specific binding of EVs [70]. EVs can also be bioengineered to increase their targetability [71]. EVs can even cross biological barriers, including the blood–brain barrier [72].

EVs may provide better therapeutic efficacy compared to liposomes which are synthetic DDS. Both EVs and liposomes have a phospholipid base [73]. However, EVs are natural, whereas liposomes are artificially synthesized [74]. As the targeting efficacy of a natural DDS is dramatically higher than that of a synthetic DDS, EVs can target the infected area more precisely than liposomes [75,76]. EVs can exhibit improved drug delivery efficiency compared with synthetic liposomes. Indeed, EVs can function as protein-ornamented phospholipid drug carriers with unique barcodes for their targets at either local or distant sites. These attributes increase target-specific functions and reduce non-specific functions of the EVs [70]. With the help of the biological traits of their cargo, EVs can reprogram their target cells [77]. EVs can also mimic the cell membrane better than liposomes [70].

### 5.1. ACE2-Modulating EVs

Due to the central role of ACE2 in COVID-19 pathogenesis, different strategies can be considered to harness EVs as an effective tool in the treatment of COVID-19. The potential strategies include EV-based DDSs, EV-based vaccines, and modulation of EV biogenesis and uptake pathways [78]. EV-based DDSs have already shown promising results in managing COVID-19 in an in vitro study where researchers have utilized recombinant human ACE2 (rhACE2) as decoy receptors to inhibit SARS-CoV-2 entry into host cells. They found that in comparison with the vesicle-free recombinant human ACE2, recombinant ACE2-expressing EVs are 135-fold more efficient in preventing the attachment of the RBD of a viral spike protein and are 60 to 80-fold more effective in preventing infections by both original and pseudotyped SARS-CoV-2 [79].

**Figure 3:**
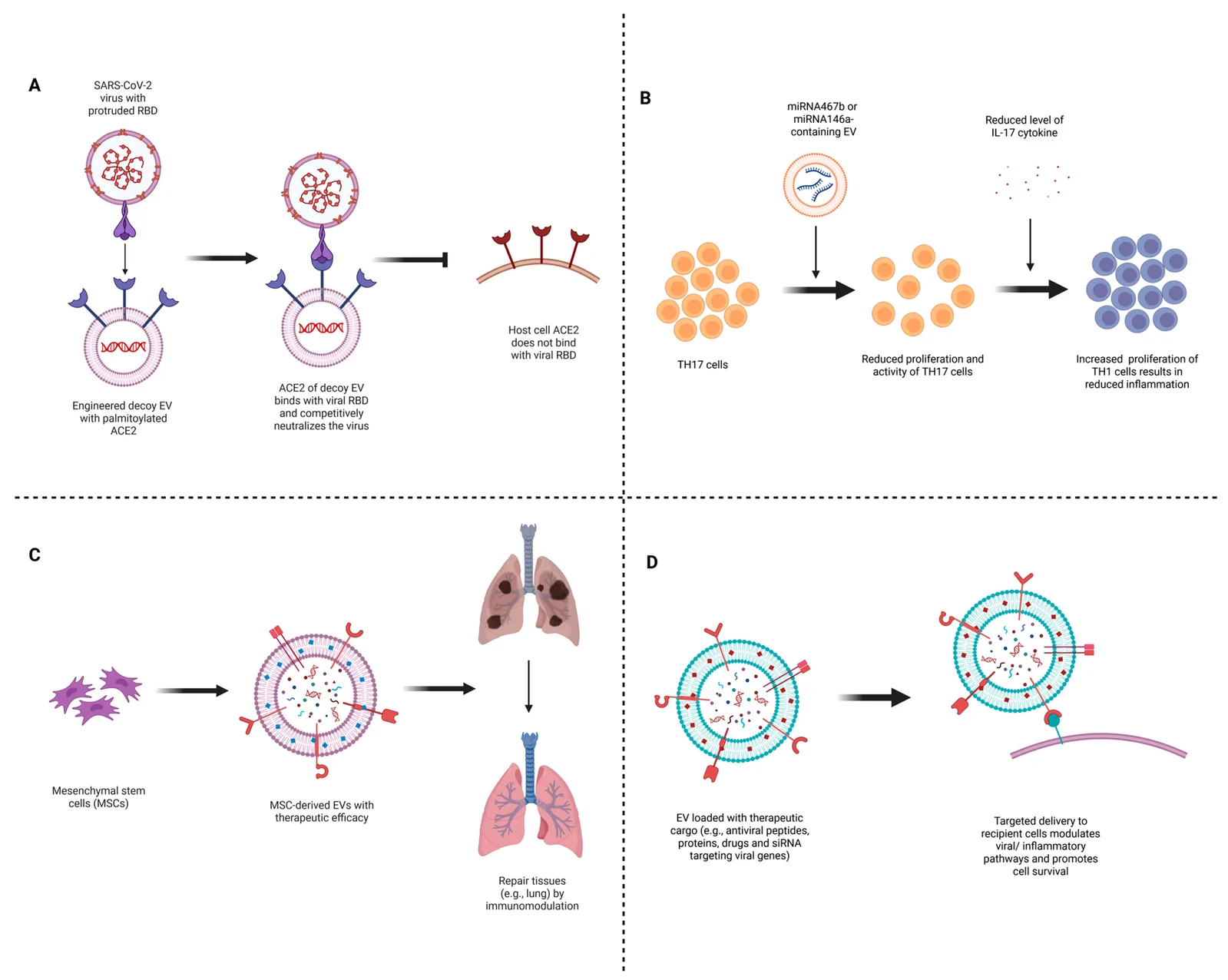
Therapeutic potential of EVs for COVID-19: (**A**) Schematic diagram of how ACE2 of engineered decoy EV binds with viral RBD and competitively neutralizes the virus to hinder viral dissemination. (**B**) Schematic diagram of how EVs carrying regulatory nucleic acids, including miRNA467b or miRNA146a, reduce the proliferation and activity of TH17 cells and increase the proliferation and activity of TH1 cells by reducing the level of proinflammatory cytokine IL-17 and ultimately reduce hyperinflammation. (**C**) Schematic diagram of how MSC-derived EVs with therapeutic efficacy can reduce hyperinflammation and result in lung tissue repair. (**D**) Schematic diagram of how EVs loaded with therapeutic cargo (e.g., antiviral peptides, proteins, drugs and siRNA targeting viral genes) can modulate viral and inflammatory pathways to increase cell survival. Created in BioRender. Dutta, S. (2026) https://BioRender.com/otej18n.

Levels of EV-ACE2 interaction are determined by their protein palmitoylation [56]. Palmitoylation and depalmitoylation at specific residues are crucial for ACE2 membrane targeting and its associated secretion into EVs. ACE2 is S-palmitoylated by an enzyme, zinc finger DHHC- type palmitoylated transferase 3 (ZDHHC3), at Cys141 and Cys498 residues. In contrast, ACE2 is depalmitoylated by another enzyme, acyl protein thioesterase 1 (LYPLA1) [56]. Palmitoylated ACE2-carrying EVs are 10 times more efficient in reducing viral infection than regular ACE2-carrying EVs. In vitro and in vivo studies have shown that Palmitoylated ACE2-carrying EVs can effectively reduce the interaction between the RBD of SARS-CoV-2 and ACE2 expressed on host cells by serving as competitive decoy receptors that sequester SARS-CoV-2 particles (Figure 3A). Hence, these engineered EVs can inhibit viral entry into host cells, subsequent infection, and viral dissemination of both authentic and pseudotyped SARS-CoV-2. Collectively, these findings suggest that palmitoylated ACE2-carrying EVs may represent a promising therapeutic strategy for the treatment of SARS-CoV-2 or similar viral infections [56].

Despite these promising findings, ACE2-modulating EVs remain at the preclinical stage, with current evidence derived from in vitro studies and animal models rather than human clinical trials [79]. Important translational challenges include optimizing ACE2 loading and surface density, maintaining EV stability and biological activity during manufacturing and storage, establishing scalable and reproducible production, and determining the safety, biodistribution, and potential off-target effects of engineered EVs [80]. Further studies are also required to determine whether this approach retains efficacy against emerging SARS-CoV-2 variants and other viruses before clinical evaluation can be considered [79].

### 5.2. TH17 Cell-Targeting EVs

EVs possess significant immunomodulatory properties and can regulate immune responses through antigen presentation, T cell activation, and inflammatory signaling [81,82,83]. For example, dendritic cell-derived tolerogenic EVs can increase Foxp3^+^ regulatory T-cell responses and suppress inflammation [82]. Regulatory T cell-derived EVs possess miRNAs that can interact with dendritic cells to modulate immune responses. They also enhance the tolerogenicity of the EVs by increasing anti-inflammatory cytokine IL-10 secretion and reducing proinflammatory cytokine IL-6 production [81]. Moreover, lncRNA-enriched EVs can promote the targeting of virus-infected cells by cytotoxic T-cell reactivation [62].

The critical involvement of EVs in cell-specific immunomodulation has already been shown in the pathogenesis of COVID-19. During the early stage of COVID-19, increased T-helper 17 (TH17) responses result in reduced T-helper 1 (TH1) responses and subsequently result in the upregulation of proinflammatory cytokine IL-17 (Figure 3B). IL-17 cytokine overexpression is critically important in COVID-19 pathogenesis as it enables parenchymal damage and ultimately leads to acute respiratory failure. TH17 cells are crucial mediators in the initiation of hyperinflammation during COVID-19, and targeting TH17 cells can be a potential way to effectively treat COVID-19 [62,84].

EVs can shuttle specific nucleic acids to target specific signaling cascades and, thus, can help to reduce immune reactions. For example, EV-associated regulatory nucleic acids, such as miRNA467b or miRNA146a, can suppress the proliferation and activity of TH17 cells (Figure 3) [62,85,86]. This approach can promote the proliferation and activity of TH1 cells by downregulating the expression of proinflammatory cytokine IL-17, thereby attenuating hyperinflammatory responses associated with severe COVID-19 [62]. It can be a promising therapeutic strategy to manage COVID-19 and potential future viral pandemics.

However, TH17-targeting EV strategies remain at the preclinical stage and have not yet been evaluated as clinical therapies for COVID-19 [87]. Although EV-associated regulatory miRNAs have demonstrated the ability to modulate Th17/Treg-associated immune responses in experimental models, the therapeutic efficacy, cell-specific targeting, cargo stability, and appropriate dosing of engineered or naturally derived EVs require further validation [87,88]. In addition, the complexity of immune responses during COVID-19 highlights the need to achieve sufficient immunomodulation without compromising antiviral host defenses [87].

### 5.3. MSC-Derived EVs

Mesenchymal stem cell (MSC)-derived EVs have shown therapeutic potential in inflammatory lung diseases, including ALI and ARDS, in both preclinical studies and Phase I/II clinical trials (Figure 3C) [89,90,91]. In 2020, a nonrandomized cohort study was conducted to evaluate the safety and efficacy of ExoFlo, an allogeneic bone marrow MSC-derived EV-based investigational therapy, in patients hospitalized with severe COVID-19 [91]. Following a single dose of intravenous ExoFlo, patients demonstrated reduced hyperinflammatory responses, restored immune homeostasis, and improved oxygenation without any significant side effects [91].

The aforementioned study laid the foundation for a multicenter, double-blinded, randomized, placebo-controlled Phase II clinical trial, EXIT COVID-19, to evaluate ExoFlo in patients with COVID-19-associated moderate-to-severe ARDS [92]. ARDS is particularly important in the context of COVID-19 as ARDS develops in up to 81% of COVID-19 patients requiring intensive care [93]. The study demonstrated favorable safety profiles and suggested potential therapeutic efficacy, including reduced mortality, shortened time to hospital discharge and increased ventilation-free days, though larger studies remain necessary to establish definitive efficacy [92].

Currently, a Phase III clinical trial, EXTINGUISH ARDS, is evaluating the safety and efficacy of intravenous ExoFlo administration in hospitalized patients with moderate-to-severe ARDS [94]. Besides COVID-19-associated ARDS, MSC-derived EVs may also be effective in treating a wide range of inflammatory diseases such as non-COVID-19 ARDS, chronic obstructive pulmonary disease, autoimmune diseases and sepsis [91]. Collectively, these studies highlight the growing translational potential of MSC-derived EVs as immunomodulatory therapeutics for severe inflammatory diseases.

Unlike the other strategies discussed above, MSC-derived EV therapy has progressed into human clinical investigation [92]. ExoFlo has been evaluated in a Phase II randomized, placebo-controlled clinical trial in patients with COVID-19-associated respiratory failure, while a Phase III randomized, double-blind, placebo-controlled trial (EXTINGUISH ARDS; NCT05354141) is currently recruiting patients with moderate-to-severe ARDS [92,94]. Nevertheless, clinical translation remains dependent on establishing definitive efficacy and long-term safety in adequately powered trials, together with consistent EV manufacturing, characterization, dosing, and quality control [95]. Thus, although MSC-derived EVs have advanced substantially beyond the preclinical stage, they remain investigational and are not yet established as standard therapy for COVID-19 or ARDS [92,95].

### 5.4. Antiviral Peptide-Conjugated EVs

An amphibian peptide, AR-23, has recently demonstrated promising antiviral efficacy against the early stages of viral infection by enveloped DNA and RNA viruses, including SARS-CoV-2 [96]. AR-23 is a 23-amino-acid amphipathic α-helical peptide [97,98]. It is derived from the skin secretions of a Japanese frog, *Rana tagoi*, and it has effective antiviral activity against both alphacoronavirus (HCoV-229E) and betacoronavirus (SARS-CoV-2) [96]. In vitro studies showed that the AR-23 peptide achieved 60% viral inhibition at 25 µM concentration during co-treatment assays and 50% inhibition at 12.5 µM concentration during pre-treatment assays (Figure 3D). AR-23 peptide can also reduce the expression of viral spike (S) and nucleocapsid (N) proteins in a dose-dependent manner, suggesting inhibition of viral entry and replication [96].

Mechanistically, the AR-23 peptide may promote viral inactivation by disrupting the viral envelope and detaching S proteins from the viral surface [96]. Given their promising biocompatibility and efficient cargo-delivery properties, EV-based DDS may represent effective platforms for transporting antiviral peptides, such as AR-23, to virus-infected cells. Consequently, EV-conjugated antiviral peptides may provide a potential therapeutic strategy for COVID-19 and other enveloped viral infections. However, further studies are necessary to evaluate the safety, efficacy, targeting capacity, and translational applicability of EV-based antiviral peptide delivery systems.

Despite the antiviral activity of AR-23, the proposed EV-conjugated AR-23 strategy remains at the preclinical/conceptual stage and has not entered clinical trials for COVID-19 [96]. Current evidence supports the antiviral activity of the free peptide, whereas the therapeutic efficacy and safety of its EV-mediated delivery have yet to be established in appropriate animal models and human studies [80,96]. Key translational challenges include reproducible peptide loading or surface conjugation, preservation of EV and peptide activity, efficient delivery to infected tissues, pharmacokinetic and biodistribution characterization, and evaluation of potential toxicity or immunogenicity [80]. Therefore, further preclinical validation is required before clinical development of EV-conjugated antiviral peptides can be considered [80].

### 5.5. Platelet-Derived EVs

Platelets play a critical role in the pathogenesis of COVID-19 by contributing to thromboinflammatory responses [99]. Platelet interaction with neutrophils causes inflammatory responses leading to multiorgan failure in severe COVID-19 [100]. Studies have shown that COVID-19 patients generate phenotypically hyperactive platelets associated with increased release of platelet granules and extracellular vesicles [99]. In COVID-19 patients, systemic inflammation promotes the release of increased levels of platelet-derived EVs [99]. Inflammatory conditions during COVID-19 also facilitate the systemic release of platelet-derived EVs, which can further modulate coagulation, endothelial dysfunction, and immune responses [100].

As platelet-derived EVs are critically involved in thromboinflammatory pathways, they have the potential to act as therapeutic targets for severe COVID-19 [101]. Given their involvement in thromboinflammatory pathways, platelet-derived EVs may represent potential therapeutic targets for attenuating coagulopathy, immunothrombotic complications, and vascular inflammation associated with severe viral infections. Therapeutic strategies aimed at reducing platelet activation or inhibiting the generation and prothrombotic signaling of platelet-derived EVs may help attenuate thromboinflammation and vascular complications associated with severe COVID-19 [101]. Hence, platelet-derived EVs may offer feasible translational opportunities for the development of therapeutic strategies against COVID-19 and potentially future viral pandemics.

Although platelet-derived EVs are increasingly recognized as contributors to COVID-19-associated thromboinflammation and potential therapeutic targets, strategies specifically designed to selectively inhibit or eliminate pathogenic platelet-derived EVs have not yet progressed to clinical trials [101]. Current challenges include identifying disease-associated platelet-EV subpopulations, selectively targeting pathogenic EV generation or signaling without impairing physiological platelet and hemostatic functions, and establishing reliable methods for EV quantification and functional assessment [95,101]. Consequently, therapeutic targeting of platelet-derived EVs remains primarily a preclinical concept, and further mechanistic and translational studies are required to establish its safety and therapeutic feasibility [101].

## 6. Diagnostic Potentials of EVs in COVID-19

Over the past decade, EVs have been used as both diagnostic and prognostic noninvasive biomarkers in different liquid biopsies [69]. Currently, EVs are emerging as promising biomarker candidates for various pulmonary diseases that lack reliable predictive diagnostic biomarkers. The role of EVs as mediators of intercellular transfer of miRNAs in pulmonary cellular communication has already been established. EVs can be critically involved in both the physiology and pathology of human lungs by coordinating and releasing homeostatic and pathological signals, respectively [102]. Due to their critical involvement in pulmonary disease processes, lung-derived EVs are evolving as promising biomarkers for various pulmonary diseases. These EVs are found in abundance in different pulmonary fluids, including epithelial lining fluid, pleural fluid, edema fluid, mucus, pulmonary circulation, lymph, etc. [77].

A proteomics-based comparison between EVs from SARS-CoV-2-infected patients and those from healthy subjects identified certain EV-associated biomarkers of COVID-19. Among these markers, the coat complex subunit beta 2 (COPB2) has emerged as a promising candidate for distinguishing mild from severe COVID-19. SARS-CoV-2 RNA has also been detected within EV cargo, suggesting a potential role for EVs in viral dissemination while highlighting their utility as indicators of infection. Moreover, markers of COVID-19 are found in platelet-derived EVs. The abundance of these markers increases with disease severity. Therefore, platelet-derived EVs can assist in diagnosing not only the presence of COVID-19 but also the severity of the disease. Key markers for platelet-derived EVs with promising potentials are HMGB1^+^, CD41a^+^/CD31^+^, CD41^+^/Annexin V^+^ CD41^+^, CD45^+^, P-Selectin^+^, and E-Selectin^+^ [103]. A recent study has also identified platelet-derived EVs as a hallmark of SARS-CoV-2 infection, further confirming the role of platelet-derived EVs as promising biomarkers of COVID-19 [104].

As EVs are critically involved in the pathogenesis of COVID-19, such as providing receptors for viral entry into the host cell and dissemination of the infection, EVs can be considered promising biomarkers for COVID-19 diagnosis [105]. A study found that quantification and phenotypic characterization of plasma EV levels can have tremendous diagnostic and monitoring value in COVID-19 [106]. Several additional EV-associated biomarkers may serve as promising tools for COVID-19 diagnosis, including SARS-CoV-2-specific S1+ serum EVs, core histone macroH2A.1 (MACROH2A1) in serum EVs, monosialodihexosyl ganglioside (GM3)-enriched exosomes, and CD142-containing EVs [69,107,108,109].

Taken together, the inherent biological and molecular characteristics of EVs provide considerable potential for improving the diagnosis, prognosis, and monitoring of viral infections [15]. EV-associated proteins, lipids, and nucleic acids offer advantages as candidate biomarkers, including accessibility through liquid biopsy, relative stability in biological fluids, and the ability to reflect dynamic host–pathogen interactions [10]. However, most reported COVID-19 EV biomarkers remain investigational [110]. Their clinical utility is limited by heterogeneous patient populations, differences in EV isolation and characterization, variable analytical platforms, and the absence of universally validated reference ranges or diagnostic thresholds [34]. Consequently, sensitivity, specificity, reproducibility, and clinical performance of individual EV biomarkers have not yet been consistently established across independent cohorts [111,112]. Large prospective studies using standardized pre-analytical and analytical workflows are needed before individual EV biomarkers can be considered clinically validated diagnostic or prognostic tests [34].

Collectively, current evidence indicates that EVs possess considerable potential as therapeutic platforms and diagnostic biomarkers for COVID-19, but the maturity of these applications differs substantially. Table 2 provides a summary of the major EV-based therapeutic and diagnostic strategies, highlighting their biological rationale, current stage of evidence, principal advantages, and remaining translational challenges.

## 7. Implications for Future Viral Pandemics

Improving preparedness for future pandemics remains a major global priority. Ideally, future pandemic preparedness should possess platform-based and adaptable therapeutic approaches capable of rapid modification to target diverse viral mechanisms [114]. Valuable mechanistic and epidemiological insights can be gained from studying previous pandemics [115]. Experts have hypothesized that the new pandemic is most likely to be caused by a respiratory virus [116]. Historical trends indicate that zoonotic viruses, particularly RNA viruses with high mutation rates and broad host ranges, will likely cause more frequent and severe pandemics in the coming years [117,118]. Researchers have recognized seven viral families that have the highest possibility of causing the next pandemic, but no treatment is available for the majority of the high-risk viral families [119]. Viral families, such as coronaviruses and influenza viruses, are particularly concerning due to their capacity for rapid transmission, evolution, and immune evasion [114]. Other respiratory pathogens, including respiratory syncytial virus and emerging paramyxoviruses, also interact extensively with host–cell trafficking machinery and innate immune pathways [120,121]. In addition, antigenic drift and recombination can develop viral variants with altered pathogenicity and improved resistance to existing interventions [122]. Increasing human–animal interface, global travel, rapid urbanization, and climate change can potentially surge the spread of evolving pathogens [123]. These shared features create a rationale for exploring host-directed and platform-based technologies.

Epidemiologic studies have found that viral pandemics, historically, have similarities in their nature and they follow a similar characteristic pattern [115,124]. Though common biological and pathogenic characteristics among viral pandemics may enable the application of EV-based therapeutic and diagnostic strategies developed for COVID-19 to future viral outbreaks [125], we have to recognize that mechanisms identified for SARS-CoV-2 may not automatically be extrapolated to other viruses. As key challenges of upcoming pandemics will be the early detection of evolving pathogens and the rapid deployment of targeted therapeutics [114], advances in high-throughput sequencing, molecular surveillance, and systems biology can play critical roles in the early diagnosis of viral pathogens [126]. Integrating mechanistic insights from host–virus interactions with scalable therapeutic and diagnostic technologies can be essential to address the impact of emerging viral pathogens [114].

EV biology may provide such a platform in this context because EVs participate in viral entry, intracellular trafficking, immune modulation, and intercellular transfer of nucleic acids and proteins across diverse viral infections. Receptor-decoy EVs, targeted cargo delivery, and immunomodulatory EVs could in principle be adapted to viruses that use conserved host receptors or inflammatory pathways [14]. Moreover, EV-associated molecular signatures offer opportunities for early, non-invasive detection and real-time monitoring of disease progression across different viral infections [14]. However, these applications remain largely conceptual for future pathogens and will require pathogen-specific validation, rapid manufacturing, standardized characterization, and adaptable regulatory pathways [127,128]. The value of EVs for pandemic preparedness therefore lies less in a universal EV therapy than in their potential to serve as modular platforms that can be re-engineered as new viral threats emerge.

A key advantage of EV-based platforms for future pandemic preparedness may be their modular adaptability to pathogen-specific requirements [129]. Established EV platforms could potentially be re-engineered by modifying surface molecules or molecular cargo to display pathogen-binding molecules, deliver antiviral nucleic acids, proteins, peptides, or small molecules, or carry immunomodulatory cargos [130]. EV-associated molecular signatures could likewise be adapted for pathogen-specific disease detection and monitoring [131]. Such approaches could retain a common framework for EV production and characterization while allowing therapeutic or diagnostic components to be modified for individual viral threats, potentially facilitating adaptation to genetically diverse or emerging viruses [129,130]. However, translation of this concept will require suitable viral or host targets, efficient and reproducible EV engineering, preservation of EV functionality, pathogen-specific validation of efficacy and safety, and scalable manufacturing with standardized quality control [34,132]. Because viral structure, tropism, replication strategies, and host responses differ among pathogens, the optimal EV strategy will likely need to be tailored to the specific viral threat [133]. Ideally, EVs should be considered as potentially adaptable pandemic-response platforms rather than as a universal therapeutic solution [129,130,132].

Collectively, EV-based technologies could complement established surveillance, diagnostics, vaccination, and antiviral-development programs by providing adaptable tools for host-directed intervention and liquid-biopsy monitoring. The mechanistic insights gained from EV–virus interactions in COVID-19 may highlight their broader relevance in combating emerging viral pathogens. Hence, EVs may help in improving preparedness and response to future viral pandemics. Importantly, advances in EV engineering and analytical technologies are expected to enhance specificity, scalability, and clinical applicability [134]. However, their broader utility will depend on whether EV products can be manufactured reproducibly, characterized rapidly, and deployed at scale during an outbreak [128]. Thus, the relevance of EVs to future pandemics should be viewed as a promising preparedness opportunity rather than a clinically established solution.

## 8. Major Challenges and Potential Limitations

Despite their advantages as a potential DDS, EV-based therapeutic platforms are subject to substantial challenges that constrain their therapeutic outcomes [12]. Variability in the final product is a major obstacle to EV-based therapeutic platforms [9]. The isolation and purification processes of EVs are not standardized yet [9]. The characterization process of some EVs is also not well-established [135]. As a result, these factors may lead to significant variations in the final yield. To establish EV-based therapeutic platforms as a suitable DDS, this concern must be addressed on a priority basis. Otherwise, it can lead to varied therapeutic outcomes and make the feasibility of EVs questionable as a potential DDS [70].

Another bottleneck is that there is very limited room for loading exogenous drugs into EVs [71]. The nano-sized vesicles contain miscellaneous contents from their cells of origin; thus, inadequate room is available in EVs for the loading of exogenous drugs [71]. This can substantially limit our ability to load the required amount of cargo in EVs for therapeutic purposes. Moreover, the clinical-grade production of EVs is insufficient, considering the high requirement to meet the global demand of pharmaceutical and biomedical companies [71]. For clinical-grade production, a consensus is also required regarding the cell type or source of EVs [136] so that it can ensure consistent product quality and compliance with regulatory and ethical standards [137]. The targeting specificity of EVs, although promising, still needs refinement to ensure drugs are delivered to the right cells without off-target effects [70].

Beyond production scale, establishing consistent manufacturing and quality control remains a major challenge for clinical translation of EV-based therapeutics [95]. Variations in the source and culture conditions of EV-producing cells, isolation and purification methods, and storage conditions can affect EV yield, composition, purity, potency, and stability, making batch-to-batch consistency difficult to achieve [34,95]. Comprehensive characterization of EV preparations, including assessment of particle concentration, size distribution, identity, purity, cargo composition, and biological activity, is therefore necessary to establish reproducible product quality and safety [34]. In addition, the regulatory framework for EV-based therapeutics remains complex and varies across jurisdictions, partly because of differences in EV sources, manufacturing processes, composition, and proposed mechanisms of action [138]. Large-scale production will consequently require scalable and closed manufacturing processes, defined raw materials, validated purification procedures, appropriate release criteria, and robust quality-control systems compatible with good manufacturing practice (GMP) [95,139]. These requirements represent substantial barriers to translating promising preclinical EV platforms into reproducible, clinically deployable products [95,139].

Furthermore, while EVs show promise for diagnostics, the development of reliable and widely applicable EV-based diagnostic tests for COVID-19 and other diseases remains a work in progress [70]. Lastly, the biological mechanisms through which EVs impact viral infection, immune modulation, and drug delivery are not yet fully understood, making it difficult to optimize EV-based therapies and diagnostics [10]. Addressing these limitations is crucial for advancing EV-based tools for the treatment and diagnosis of COVID-19 and future viral pandemics.

## 9. Conclusions

**Figure 4:**
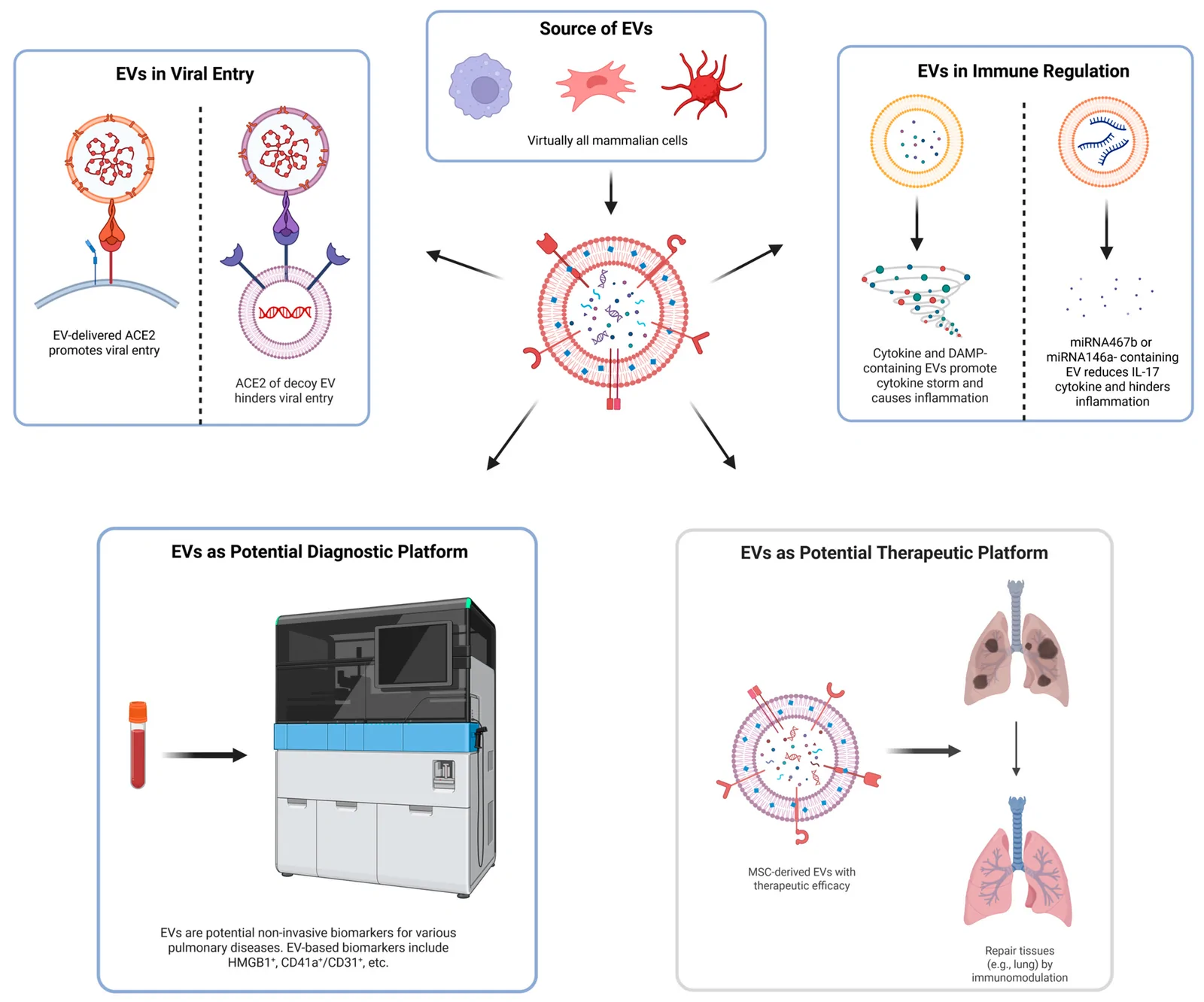
Schematic diagram of the major areas that are covered in this review. It summarizes the biogenesis, structure, molecular mechanisms, and therapeutic and diagnostic potentials of EVs as described in this review. Created in BioRender. Dutta, S. (2026) https://BioRender.com/2yvmswr.

Extracellular vesicles (EVs) have emerged as key regulators of viral pathogenesis and represent promising platforms for therapeutic and diagnostic applications in COVID-19 (Figure 4). Through their ability to modulate viral entry, immune responses, and inflammatory signaling, EVs offer unique opportunities for targeted intervention. Engineered EVs, including viral decoys, drug delivery vehicles, and immunomodulatory agents, further expand their translational potential. In addition, EV-associated molecular signatures provide valuable tools for non-invasive diagnosis and disease monitoring. Insights into EV–virus interactions may also help develop strategies to address future viral pandemics. However, challenges such as difficulty in loading adequate cargo, limited scalability, and the need to improve targeting efficiency must be addressed to enable clinical translation. Overall, continued advances in EV biology and single-vesicle technologies are expected to drive the development of next-generation precision therapeutics and diagnostics for viral diseases. Nevertheless, several key questions remain regarding the reproducibility and potency of EV-based products, their in vivo biodistribution, the optimal dosing strategies, and their long-term safety and immunogenicity. Addressing these uncertainties, together with establishing standardized manufacturing, characterization, and regulatory criteria, will be essential before EV-based therapeutics and diagnostics can achieve broad clinical use.

## Figures and Tables

**Table 1:** Molecular mechanisms through which extracellular vesicles regulate SARS-CoV-2 infection and host responses.

Mechanistic Axis	Representative EV Cargo/Components	Biological Functions	Translational Significance	Representative References
Immune regulation	Clusters of viral DNA, RNA, and other viral particles	Bidirectional regulation of viral infection based on the cargo.	Precision EV engineering	[13,26,48,61,62]
ACE2-dependent interactions	ACE2-positive EVs	ACE2-bearing EVs can increase susceptibility or function as competitive decoy receptors depending on the context.	Basis for engineered ACE2-decoy therapeutics	[16,49,55,56]
Viral activation and trafficking	TMPRSS2, Rab GTPases, TSG101, ALIX, ESCRT	EVs can deliver proteases to activate viral spike protein, and EV biogenesis shares common viral entry and trafficking pathways.	Targets EV biogenesis	[16,57,58]
Inflammatory signaling	TNF-α, IL-6, HMGB1	Amplifies NF-κB-mediated cytokine production and results in cytokine storm.	Anti-inflammatory EVs	[63,64,65]
Inflammasome activation	mtDNA, viral RNA, ROS-associated signaling molecules	Promotes NLRP3 activation that triggers caspase-1 activation and pyroptosis and results in lung injury.	Modulates pathogenic cargo	[66,67,68]

**Table 2:** Current and emerging extracellular vesicle-based therapeutic and diagnostic strategies against COVID-19.

EV Strategy	Biological Rationale	Current Evidence	Major Advantages	Key Limitations	Representative References
ACE2-modulating EVs	Neutralize SARS-CoV-2 through receptor-decoy activity.	Robust preclinical	High specificity; targeted antiviral action	Manufacturing and scalability	[55,56,78,79]
TH17 cell-targeting EVs	Deliver anti-inflammatory regulatory cargo.	Preclinical	Cell-selective immune modulation	Optimization of cargo loading	[62,81,82,83,84,85,86]
MSC-derived EVs	Suppress excessive inflammation and promote tissue repair through bioactive cargo.	Preclinical/early clinical	Low immunogenicity; high regenerative potential	Standardization is required	[89,90,91,92,93,94]
Antiviral peptide-conjugated EVs	Facilitate viral inactivation by damaging the viral envelope and detaching S proteins.	Mechanistic/observational	High therapeutic potential	Safety, efficacy, targeting capacity, and translational applicability need to be evaluated	[96,97,98]
Platelet-derived EVs	Inhibit platelet activation/prothrombotic signaling of platelet-derived EVs	Mechanistic/observational	Effectiveness in thromboinflammation and vascular complications	Safety, efficacy and stability need to be assessed	[99,100,101]
Drug-/RNA-/protein-loaded EVs	Improve targeted delivery of antiviral agents.	Preclinical	Excellent biocompatibility; high target-specificity; low non-specific function	Production scale and loading efficiency	[70,71,72]
EV biomarkers	Proteins, RNAs and lipids reflect disease status.	Multiple clinical	Minimally invasive; stable; represent host–pathogen interaction; early disease detection; prognostic assessment; patient stratification; therapeutic monitoring	EV isolation, characterization, and molecular profiling	[10,15,20,69,77,102,103,104,105,106,107,108,109,113]

## Data Availability

No new data were created or analyzed in this study. Data sharing is not applicable to this article.
